# Elucidation of the mechanism of action of Runyan Mixture in the treatment of pharyngitis using a network pharmacological approach

**DOI:** 10.1097/MD.0000000000032437

**Published:** 2022-12-23

**Authors:** Huihui Zhang, Yingpeng Tong, Yinzhi Jin, Guoyun Cai, Zhenxin Li, Xinling Pan

**Affiliations:** a Traditional Chinese medicine pharmacy, Affiliated Dongyang Hospital of Wenzhou Medical University, Jinhua, China; b College of Pharmaceutical Sciences, Taizhou University, Taizhou, China; c Department of Biomedical Sciences Laboratory, Affiliated Dongyang Hospital of Wenzhou Medical University, Jinhua, China.

**Keywords:** anti-inflammation, mechanism of action, network pharmacology, pharyngitis, Runyan mixture

## Abstract

This study aimed to elucidate the mechanism of action of Runyan Mixture in treating pharyngitis using a network pharmacological approach. The active components of the Runyan Mixture were obtained from the traditional chinese medicine systems pharmacology database and evaluated using Lipinski’s rules. The SwissTargetPrediction database was used to predict the action targets of the Runyan Mixture, and a protein-protein interaction network was constructed using the STRING database. Moreover, the anti-inflammatory effect of Runyan Mixture was validated in vitro using the lipopolysaccharide induced inflammation in macrophages. The Runyan Mixture was the liquid preparation from 8 traditional Chinese medicine. A total of 89 types of active components, 53 core targets, and 98 signaling pathways (*P* < .001) were identified for the Runyan Mixture. The main action targets were EGFR, MAPK1, AKT1, PIK3CA, NFKB1, SRC, TNF, MAPK8, MET, and PTGS2. Among the identified signaling pathways, 20 were associated with microbial infection and 24 were related to the immune-inflammatory response. Experimental results in vitro showed that Runyan Mixture could significantly inhibit the expression of interleukin-1, interleukin-6, and tumor necrosis factor-α (*P* < .05) in macrophages by lipopolysaccharide stimulation. Based on the results of the protein-protein interaction network analysis and the anti-inflammatory effect in vitro, the efficiency of the Runyan Mixture in pharyngitis treatment could be attributed to the inhibition of the inflammatory response.

## 1. Introduction

Pharyngitis is an inflammatory immune disease. Due to air pollution, climatic changes, and other factors, the incidence rate of pharyngitis is as high as 20% to 50%, making it a common disease with a rising trend.^[1]^ Clinically, pharyngitis is mainly categorized into acute pharyngitis and chronic pharyngitis. The former is mainly caused by a bacterial infection or secondary bacterial infection after a viral infection, predominantly presenting local symptoms. The latter is a chronic inflammation of the pharyngeal mucosa, submucosal tissue, and lymphoid tissue, with the main manifestations of pharyngeal pain, itching, burning sensation, dryness, irritation, hoarseness, and sometimes nausea and vomiting.^[2]^ Antibiotics are generally used to treat acute pharyngitis, which makes it easy to develop drug resistance.^[3]^ Studies have shown that the use of antibiotics did not improve the symptoms; therefore, researchers in China and other Asian countries^[4–6]^ are trying to find alternative therapies such as using traditional Chinese medicine (TCM), which is effective in treating pharyngitis.

Runyan Mixture is a hospital liquid preparation that is obtained via boiling extraction from composed of 8 TCMs. The main medicine against inflammation of pharynx is *Solidaginis herba*, which is also assisted by *Scrophulariae radix*, *Forsythiae fructus*, and *Chrysanthemi indici Flos*. The main medicines for relieving dry cough and sputum are *Asparagi radix*, *Ophiopogonis radix*, and *Rehmanniae radix*, which have the common therapeutic effect. *Glycyrrhizae radix et rhizome*, play the role of moderating the property of the TCMS, so that all medicines of this prescription together to relieve the symptoms of pharyngitis, such as dry cough and swelling and pain in pharynx. Single or combined usages of these TCMs have been described efficient in the treatment of inflammation related diseases.^[7–9]^ Runyan Mixture has been used for the treatment of pharyngitis at our hospital for several years, and clinical observation suggested that Runyan Mixture has a significant alleviating effect (data not published).

However, the compounds of the Runyan Mixture are relatively complex, making it difficult to explain the specific mechanism of action, thereby affecting the promotion and application of TCM. Network pharmacology is a new model in drug research proposed by Andrew L Hopkins in 2007, which can be used to build and analyze complex networks of “component-target-disease” based on systems biology, multidirectional pharmacology, network biology, computer technology, and various omics technologies, to explore the relationship between drugs and diseases comprehensively.^[10–12]^ Therefore, we adopted network pharmacology in the current study to clarify the mechanism of action of Runyan Mixture in the treatment of pharyngitis and verify the anti-inflammatory effect using in vitro inflammation model.

## 2. Materials and methods

### 2.1. Screening the chemical components of Runyan Mixture

In this study, the name of the active medicinal material in the Runyan Mixture was used as the keyword, and the chemical components of each medicinal material were obtained from the traditional chinese medicine systems pharmacology database (https://old.tcmsp-e.com/tcmsp.php). The Lipinski’s Rule of Five, namely molecular weight (MWV500), lipid-water partition coefficient (AlogP < 5), number of hydrogen bond donors (Hdon < 5), number of hydrogen bond acceptors (Hat < 10), and rotational bonds (RBN < 10), was used to preliminarily screen the active components of Runyan Mixture.

### 2.2. Prediction of targets and construction of protein-protein interaction (PPI) Network

The chemical structures of the active ingredients were illustrated using ChemBioDraw Ultra 14.0, saved in SMILES format, and uploaded to the SwissTargetPrediction (http://www.swisstargetprediction.ch/) database to list the targets of the Runyan Mixture. SwissTargetPrediction is an online tool for predicting small molecule targets. In this study, the probability value for evaluating the accuracy of target prediction was set as >0.

The disease targets were retrieved from GeneCards database by the keyword of pharyngitis. The common targets between disease and Runyan Mixture were imported into the STRING database to build the PPI network. The specific parameter settings were as follows: The species was *Homo sapiens* with the confidence set to the highest value (0.700). The unconnected nodes in the network were hidden, and the other parameters were set to default values. The analysis result file was downloaded in CSV format and imported into Cytoscape 3.7.2 software. The PPI network was analyzed using the Network Analyzer tool, and the core targets of the Runyan Mixture were screened according to degree value.

### 2.3. Functional analysis and action network

The core targets were imported into the DAVID database (https://david.ncifcrf.gov/summary.jsp) to perform the gene ontology (GO) function and kyoto encyclopedia of genes and genomes (KEGG) signaling pathway enrichment analyses and *P* < .001 was considered as the criterion for screening the signaling pathways of the Runyan Mixture. Cytoscape 3.7.2 software was used to establish the network of chemical components of Runyan Mixture-their targets-signaling pathways. The nodes in the network represented the chemical components of the Runyan Mixture, their targets, and signaling pathways, which were connected by edges if the corresponding nodes showed an interaction.

### 2.4. Cell experiment validation

#### 2.4.1. Preparation of the extracted liquid from Ryan Mixture.

Runyan Mixture was a preparation of Dongyang People’s Hospital (batch number: 20201207). It was a brown to black-brown liquid with a slightly fragrant odor and sweet and slightly bitter taste. The drug liquid was centrifuged at 3000 rpm for 30 minutes, and the supernatant was filtered using a 0.22 μm filter for sterilization, followed by dilution for 5, 50, 500, 5000, and 50,000 times in 1640 complete medium (containing 10% fetal bovine serum) and storage at 4 °C for subsequent usage.

#### 2.4.2. The verification of the anti-inflammatory effect of drug solutions in vitro.

THP-1 cells were seeded into 6-well plates at a density of 1 × 10^6^ cells/well. After differentiation induced by 50 ng/mL phorbol-12-Myristate-13-Acetate for 24 hours, the cells were cultured in fresh 1640 complete medium overnight. Inflammatory stimulation was performed with 1 μg/mL lipopolysaccharide (LPS), and cells were collected without the action of drug concentration for 12 hours.

The total RNA of the collected THP-1 cells was extracted using the Trizol (Takara, Shiga, Japan) method and measured using nanodrop. About 1 μg of total RNA was reverse-transcribed to cDNA using the reverse transcription kit (Takara). The reverse transcription system of 20 μL included 4 μL 5X PrimeScript Buffer, 1 μL PrimeScript RT Enzyme Mix I, 1 μg RNA, 50 pmol Oligo dT primer, and 100 pmol random primers. The reverse transcription conditions were as follows: 37 °C for 15 minutes and 85 °C for 5 seconds. The interleukin (IL-1), IL-6, and tumor necrosis factor-α (TNF-α) mRNA levels were detected using real-time quantitative PCR (Takara) and the primer sequences are shown in Table S1, Supplemental Digital Content, http://links.lww.com/MD/I204. The quantitative PCR system (20 μL) included 10 μL TB Green Premix Ex Taq II, 1 μL upstream and downstream primers, 2 μL cDNA, and 6 μL H_2_O. Quantitative PCR was performed using a QuantStudio 5 (Thermofisher, Waltham, Massachusetts) amplification instrument, and the conditions were pre-denaturation at 95 °C for 30 seconds, and 40 cycles of amplification (95 °C for 3 seconds and 60 °C for 30 seconds). GAPDH was used as an internal reference to calculate 2^−ΔCT^. The expression of cytokines in the experimental group was compared with that in the LPS stimulation group as the control group.

### 2.5. Statistical analysis

SPSS 18.0 software (IBM, Chicago) was used for the statistical analysis of experimental data. The data were expressed as mean ± standard deviation (*X* ± S). The *T* test was used for pairwise comparison, and a *P* value < .05 was considered to be statistically significant.

## 3. Results

### 3.1. The chemical components and targets of Runyan Mixture and construction of PPI network

The work flow was showed in Figure S1, Supplemental Digital Content, http://links.lww.com/MD/I205. The names of 8 TCMs in Runyan Mixture were input and a total of 89 chemical compounds were identified based on the Lipinski’s Rule of Five. A total of 801 unique targets corresponding to the 89 compounds were listed using the SwissTargetPrediction database.

**Figure 1.**
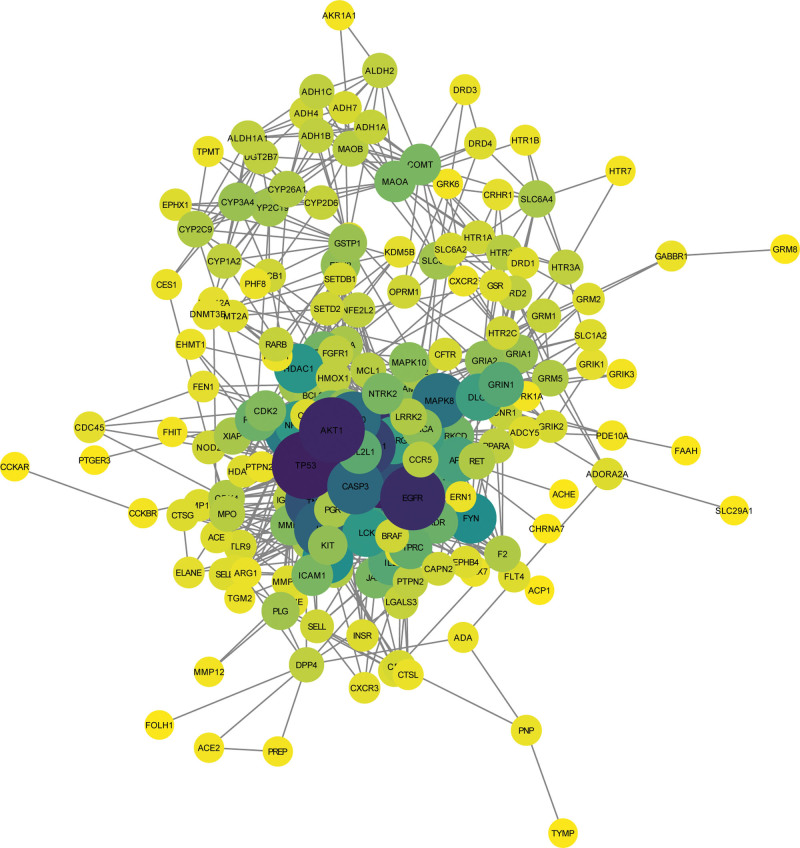
The PPI network of Runyan Mixture. The bubbles represent the core targets of the Runyan Mixture; the size of the bubbles represents the gene number involved in each pathway; the color ranged from light to dark represents *P* values changed from small to larger, PPI = protein-protein interaction.

The intersection analysis of the targets of Runyan Mixture and pharyngitis disease was conducted, and 228 intersection targets were identified. The PPI network of Runyan Mixture contained 207 nodes and 1328 edges and the median node degree was 9 (Fig. 1). In this study, the target with a degree ≥ 18 was determined as a core target of the PPI network; and a total of 53 core targets were identified.

### 3.2. The GO functions and KEGG signaling pathway enrichment analyses of core targets

**Figure 2.**
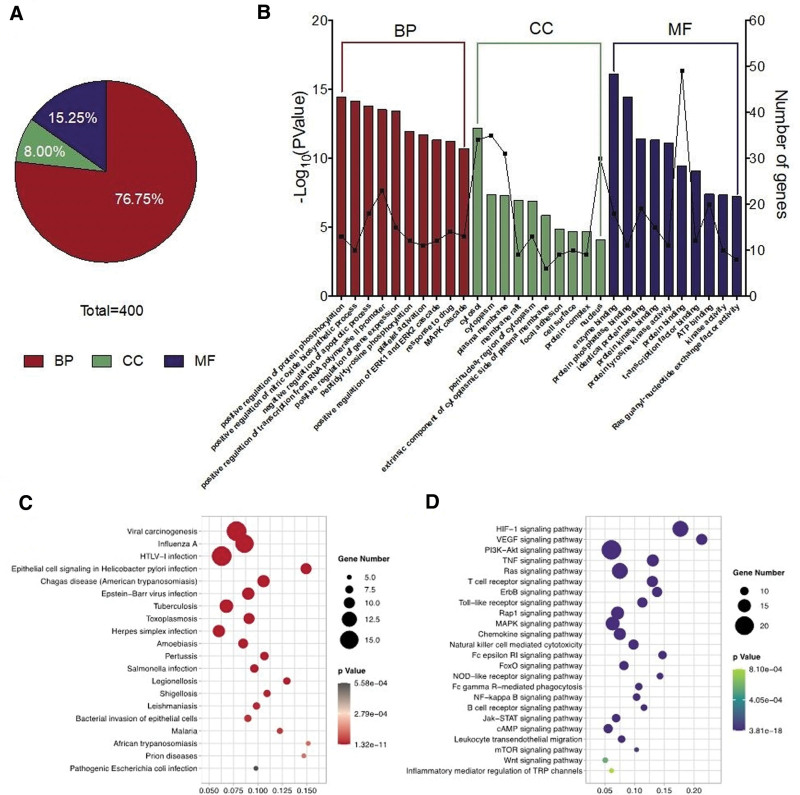
GO enrichment and enrichment of KEGG pathway of Runyan Mixture in treatment of pharyngitis. (A) Proportion chart of items of BP, CC, and MF in that of GO enrichment; (B) the top 10 items of BP, CC, and MF in GO enrichment; (C) pathway related to microbial infection; and (D) pathway related to immune-inflammatory response. BP = biological process, CC = cellular component, GO = gene ontology, KEGG = kyoto encyclopedia of genes and genomes, MF = molecular function.

GO function, KEGG signaling pathway, and disease category associated with the 53 core targets were determined using the DAVID database. As shown in Figure 2A, there were 400 GO terms (*P* < .05), including 307 terms related to biological process, 32 terms related to cellular component, and 61 terms related to molecular function, accounting for 76.75%, 8.00%, and 15.25% of the terms, respectively. The top 10 terms of biological process, cellular component, and molecular function are shown in Figure 2B. A total of 98 signaling pathways were obtained (*P* < .001) by enrichment, including 44 signaling pathways related to microbial infection (Fig. 2C) and immune-inflammatory response (Fig. 2D).

### 3.3. Construction of the compound-target-signaling pathway network

**Figure 3.**
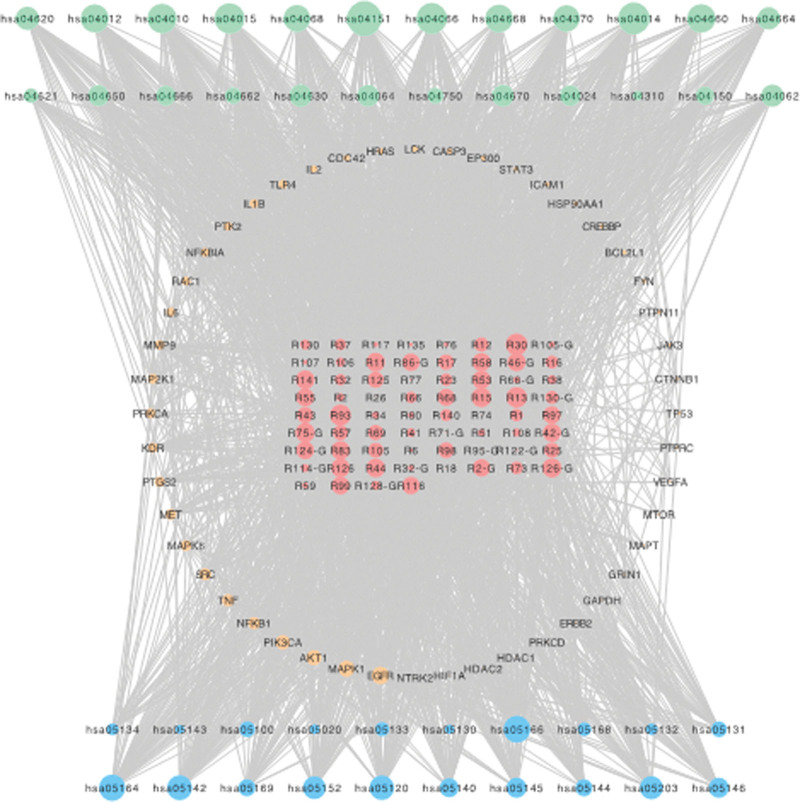
Network of compound-target-pathway of Runyan Mixture. Nodes filled in blue and green color represent the pathways related with microbial infection (pathway 1) and regulating the inflammatory response (pathway 2), respectively. Pathways with blue bubbles belong to pathway 1, which represents the pathways related with microbial infection, while pathways with green bubbles belong to pathway 2, which represents the pathways involved in regulating the inflammatory response.

The “chemical components-target-signaling pathway” network of Runyan Mixture for the treatment of pharyngitis was constructed (Fig. 3). The core targets of the network and main compounds were identified using the network topology analysis (Table 1). The active components of Runyan Mixture with higher degree values are caffeic acid (R30), medicarpin (R93), hispidulin (R75-G), kaempferol (R83), 7-hydroxycurmarin (R126-G). The structures are shown in Figure S2, Supplemental Digital Content, http://links.lww.com/MD/I206. The results indicated that Runyan Mixture may play a role in the treatment of pharyngitis mainly by acting on core targets, such as EGFR, MAPK1, AKT1, PIK3CA, NFKB1, SRC, TNF, MAPK8, MET, and PTGS2.

**Table 1:** The top 10 targets and active ingredients that affect the entire network.

Compounds	Degree	Targets	Degree	Signaling pathways	Degree
Caffeic acid (R30)	50	EGFR	40	hsa04151	79
Medicarpin (R93)	48	MAPK1	37	hsa04014	72
8-{3-Oxo-2-[(2E)-2-penten-1-yl]-1-cyclopenten-1-yl}ctanoic acid (R13)	47	AKT1	35	hsa04066	70
9S,13R-12-Oxophytodienoic acid (R15)	45	PIK3CA	33	hsa04015	69
Hispidulin (R75-G)	44	NFKB1	28	hsa04010	67
Skimmetin (R126-G)	44	SRC	28	hsa04660	65
7-Methoxy-4-methylcoumarin (R11)	43	TNF	28	hsa04012	64
α-Linolenic acid (R141)	43	MAPK8	25	hsa04668	63
Dibutyl phthalate (R44)	43	MET	25	hsa05166	61
Skimmin (R126)	43	PTGS2	24	hsa04068	61
Kaempferol (R83)	43				

### 3.4. The verification of anti-inflammation of Runyan Mixture in vitro

**Figure 4.**
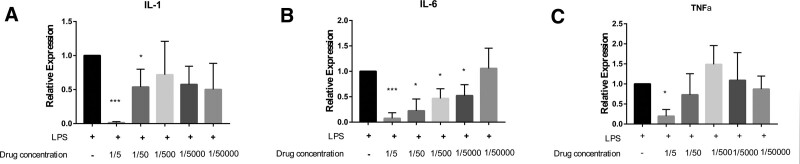
The mRNA expression levels of IL-1, IL-6, and TNF-α in LPS-stimulated THP-1 cells. *P < .05, ***P < .0001, compared to the LPS stimulation. IL = interleukin 1, LPS = lipopolysaccharide, TNF-α = tumor necrosis factor-α.

In macrophages stimulated by LPS, high concentration (5-fold dilution group) could significantly inhibit transcriptional expressions of IL-1, IL-6, and TNF-α (*P* < .05) (Fig. 4). The inhibitory effect of the drug on IL-6 was stronger than those on IL-1 and TNF-α, which was still significant even if the liquid preparation was diluted by 5000 times.

## 4. Discussion

The Runyan Mixture can effectively improve the symptoms of acute and chronic pharyngitis in clinical practice. However, its mechanism of action is not clear. Here, a combination of network pharmacology and in vitro cell experiments was performed to investigate the possible mechanisms of action of the Runyan Mixture in pharyngitis treatment.

The results of network pharmacology of Runyan Mixture showed that there were several active components in Runyan Mixture. Among them, caffeic acid is a common major active component of *Solidago decurrens*^[13,14]^ and *Chrysanthemum Indici Flos*, hispidulin is the main active component of *Chrysanthemum Indici Flos*, and kaempferol and 7-hydroxycoumarin are the main active components of *S decurrens*.^[15–17]^ The anti-inflammatory effect of kaempferol has been confirmed at the cellular level, characterized as reducing the production of pro-inflammatory cytokines such as thymic stromal lymphopoietin, TNF-α, and IL-8.^[18,19]^ Caffeic acid can clear the reactive oxygen species released by inflammatory neutrophils and macrophages, and enhance the anti-inflammatory effect of the body by reducing the expression of proinflammatory cytokines.^[20,21]^

A total of 228 targets were obtained after the intersection analysis of compound-disease targets, including 53 core targets, suggesting that the Runyan Mixture has a synergistic effect in the treatment of pharyngitis. According to the analysis results of the PPI network, the main targets of the Runyan Mixture are EGFR, MAPK1, AKT1, PIK3CA, NFKB1, SRC, TNF, MAPK8, MET, and PTGS2. Among the key signaling pathways, AKT1, MAPK1, and EGFR are also the targets of 3 iridoid glycosides from *Phlomis brevidentata H.W.Li Radix*, which is efficient in treating pharyngitis.^[22]^ PTGS2, NFKB1, and TNF have been essential in chronic pharyngitis treatment by herba Sarcandrae.^[23]^ Though the roles of other targets in pharyngitis have not been reported, they are significant in the modulation of inflammatory cytokines.^[24,25]^ The expression levels of IL-1, IL-6, and TNF-a were inhibited by Runyan Mixture in vitro results, evidently indicating its therapeutic role for pharyngitis by inhibiting the inflammatory response.

The present work also had certain limitations. The network pharmacology studies of TCM preparations do not consider the complex compounds changes and the ingredient losses from herbs during the extraction process.^[26–28]^ Therefore, further studies are required for structure identification using different methods, such as mass spectrometry molecular network. Also, further pharmacodynamic experiments are required in the future to validate the mechanism of action of the Runyan Mixture in pharyngitis treatment.

## 5. Conclusion

This study predicted the targets, mechanism of action, and signaling pathways related to the Runyan Mixture in the treatment of pharyngitis using network pharmacology. The results revealed that the mechanisms of action of the Runyan Mixture against pharyngitis were mainly attributable to pathways related to inflammatory responses. Moreover, the key active ingredients in the Runyan Mixture were caffeic acid, medicarpin, hispidulin, kaempferol, and 7-hydroxycurmarin. The in vitro experiments confirmed the anti-inflammatory effect of the Runyan Mixture by inhibiting the transcription levels of proinflammatory cytokines. These results might help clinicians understand the mechanism of action of Runyan Mixture in pharyngitis treatment.

## Author contributions

**Conceptualization:** Huihui Zhang, Yinzhi Jin, Zhenxin Li, Xinling Pan.

**Data curation:** Huihui Zhang, Yingpeng Tong.

**Formal analysis:** Guoyun Cai, Zhenxin Li.

**Funding acquisition:** Huihui Zhang.

**Investigation:** Zhenxin Li, Xinling Pan.

**Methodology:** Yingpeng Tong, Guoyun Cai, Xinling Pan.

**Software:** Huihui Zhang, Yingpeng Tong, Guoyun Cai.

**Supervision:** Yinzhi Jin, Zhenxin Li.

**Validation:** Guoyun Cai, Zhenxin Li, Xinling Pan.

**Writing – original draft:** Huihui Zhang, Xinling Pan.

**Writing – review & editing:** Yingpeng Tong, Yinzhi Jin, Guoyun Cai, Zhenxin Li, Xinling Pan.

## Supplementary Material
