# Supplementary material for: Elucidation of the mechanism of action of Runyan Mixture in the treatment of pharyngitis using a network pharmacological approach
Source: Medicine (Baltimore). 2022 Dec 23;101(51):e32437. doi: 10.1097/MD.0000000000032437 (PMC9794313; doi:10.1097/MD.0000000000032437)
Supplement: Supplementary file 1 [file medi-101-e32437-s001.pdf]

Supplementary table 1. Information of the primers used in this study

Primers		Sequences (5'-3')
GAPDH	Forward	GTCGCTGTTGAAGTCAGAGG
	Reverse	GAAACTGTGGCGTGATGG
IL-1	Forward	TGCCTTAGGGTAGTGCT
	Reverse	GCGGTTGCTCATCAGA
IL-6	Forward	AATTCGGTACATCCTCGACGG
	Reverse	GGTTGTTTTCTGCCAGTGCC
TNF-α	Forward	AGGCGGTGCTTGTTCCTC
	Reverse	GTTCGAGAAGATGATCTGACTGCC
