# Supplementary material for: Elucidation of the mechanism of action of Runyan Mixture in the treatment of pharyngitis using a network pharmacological approach
Source: Medicine (Baltimore). 2022 Dec 23;101(51):e32437. doi: 10.1097/MD.0000000000032437 (PMC9794313; doi:10.1097/MD.0000000000032437)

Supplementary figure 1. The workflow for investigating the mechanism of Runyan Mixture for pharyngitis treatment.

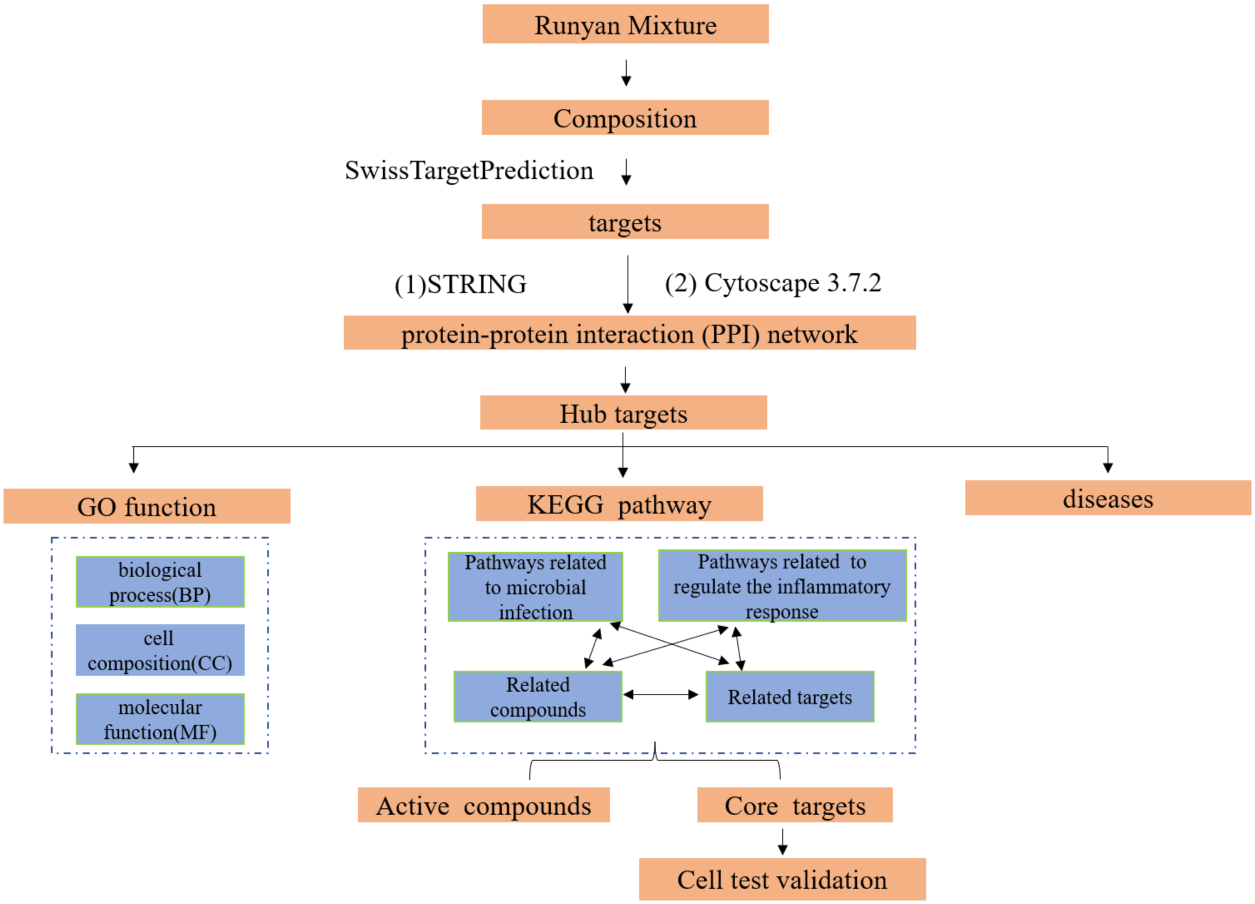

Supplement: Supplementary file 2 [file medi-101-e32437-s002.pdf]
