# Supplementary material for: Elucidation of the mechanism of action of Runyan Mixture in the treatment of pharyngitis using a network pharmacological approach
Source: Medicine (Baltimore). 2022 Dec 23;101(51):e32437. doi: 10.1097/MD.0000000000032437 (PMC9794313; doi:10.1097/MD.0000000000032437)
Supplement: Supplementary file 3 [file medi-101-e32437-s003.pdf]

Supplementary figure 2. Molecular structure of the active component of Runyan Mixture in treatment of Pharyngitis.

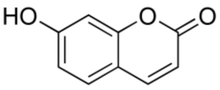
R126-G

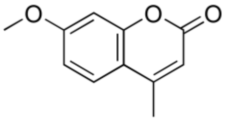
R11

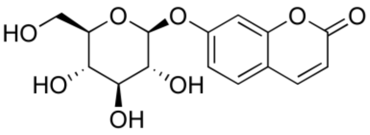
R126

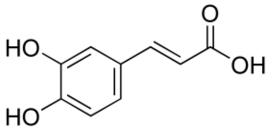
R30

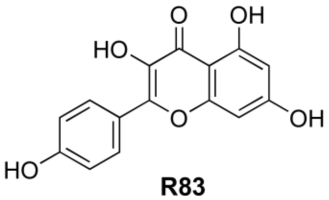
R83

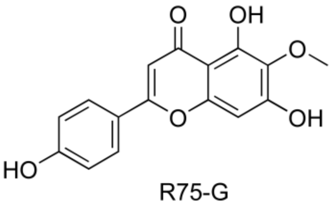
R75-G

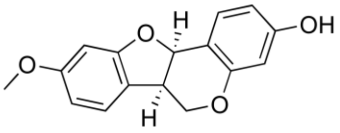
R93

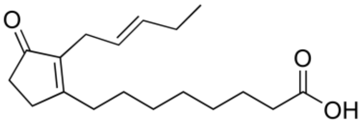
R13

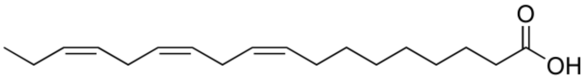
R141

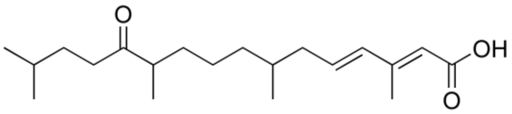
R15

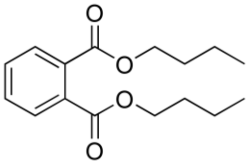
R44
